# Cytoreductive Surgery of Colorectal Peritoneal Metastases: Outcomes after Complete Cytoreductive Surgery and Systemic Chemotherapy Only

**DOI:** 10.1371/journal.pone.0122816

**Published:** 2015-03-31

**Authors:** Grégoire Désolneux, Camille Mazière, Jérémy Vara, Véronique Brouste, Marianne Fonck, Dominique Béchade, Yves Bécouarn, Serge Evrard

**Affiliations:** 1 Digestive Tumours Unit, Institut Bergonié, Bordeaux, France; 2 Clinical and Epidemiological Research Unit, Institut Bergonié, Bordeaux, France; 3 Univ. Bordeaux, Bordeaux, France; University of Florida, UNITED STATES

## Abstract

**Background:**

Cytoreductive peritoneal surgery (CRS) associated with hyperthermic peritoneal chemotherapy (HIPEC) has long been considered the standard treatment for colorectal peritoneal metastases (CPM). However, although efficacy of surgery has been demonstrated, evidence supporting HIPEC’s role is less certain.

**Method:**

Overall survival (OS), progression-free survival (PFS) and morbidity were analysed retrospectively for fifty consecutively included patients treated for colorectal CPM with complete CRS and systemic chemotherapy only.

**Results:**

Median peritoneal cancer index (PCI) was 8 (range 1-24). 23 patients had liver or lung metastases (LLM). 22 patients had synchronous CPM. 27 complications occurred (12 Grade 1/2, 14 Grade 3, 1 Grade 4a, 0 Grade 5). Median follow-up was 62.5 months (95 %CI 45.4-81.3), median survival 32.4 months (21.5-41.7). Three- and 5-year OS were 45.5% (0.31-0.59) and 29.64% (0.17-0.44) respectively. Presence of LLMs associated with peritoneal carcinomatosis was significantly associated with poorer prognosis, with survival at 5 years of 13.95% (95 %CI 2.9-33.6) vs. 43.87% (22.2-63.7) when no metastases were present (P= 0.018). Median PFS was 9.5 months (95 %CI 6.2-11.1).

**Conclusion:**

With an equivalent PCI range and despite one of the highest rates of LLM in the literature, our survival data of CRS + systemic chemotherapy only compare well with results reported after additional HIPEC. Tolerance was better with acceptable morbidity without any mortality. Extra-hepatic metastasis (LLM) is a strong factor of poor prognosis. Awaiting the results of the randomized PRODIGE trial, these results indicate that CRS + systemic chemotherapy only is a robust hypothesis to treat colorectal CPM.

## Introduction

With an occurrence rate of 10 to 13%,[1] peritoneal metastases (CPM) is the third most common colorectal metastatic disease after liver and lung metastases. CPM alone is observed in 25–35% of recurrences, but it is also frequently associated with liver and lung metastases.[2]

Since the 1990’s, an aggressive curative approach has been proposed for selected patients. This treatment includes cytoreductive surgery (CRS) combined with hyperthermic intraperitoneal chemotherapy (HIPEC).[3,4] By misnomer, HIPEC is often used in the literature to refer to the combination of surgical and properly said, HIPEC treatments (referred to as CRS + HIPEC in this paper). This combined surgery and HIPEC treatment is performed in highly specialized and tertiary care centres in which CRS +HIPEC has become the standard treatment for CPM.[5]

However, the utility of CRS+ HIPEC has not been fully demonstrated. CRS+HIPEC was formally introduced into surgical practices after a consensus paper in 2007[6] presented as based on eleven “phase 2” trials. Closer inspection of the evidence reveals that only three papers present prospective data from valid phase II trials[7–9] and the adoption of this practice may have been too hasty. A recent debate between Paul H Sugarbaker and David P Ryan,[10] opened the scientific discussion of the benefits and side effects of this procedure, even if definitive conclusions on the experimental or confirmed aspect of HIPEC are not yet possible.[11]

Unfortunately, it is impossible to separate the effects of CRS and HIPEC in the available literature. The hypothesis that CRS could be the only active part of the treatment is not new and was also raised by Verwaal et al[7] in the sole randomized study published yet: “…it cannot be excluded that the observed effect was exclusively or mainly caused by the aggressive cytoreduction alone” (p.3742). Indeed, HIPEC was initially attempted after incomplete CRS, but therapeutic efficacy was not demonstrated.

Progressively, doubts regarding the efficacy of HIPEC have been raised, and indications for HIPEC have been consecutively reduced and finally restricted to cases where surgery can remove all macroscopic disease. At this stage, HIPEC efficacy is strictly obscured by efficacy of CRS. Given this current inability to individually identify the effects of CRS and HIPEC, a randomized trial addressing the question of HIPEC efficacy directly was launched (Cytoreductive surgery with or without HIPEC for colorectal peritoneal metastases; NCT 00769405). The study is just finishing inclusions and survival results will not be available for sometime.

The primary aim of this study was thus to test in a single centre, the hypothesis of the futility of HIPEC in colorectal carcinomatosis by removing HIPEC from the treatment approach and reporting data after CRS and chemotherapy only. It is, to our knowledge, the first consecutive series of patients treated by complete CRS (CC0) plus systemic chemotherapy, challenging the traditional additional HIPEC treatment. Secondary aims were to compare survivals between metastatic and non-metastatic patients and between patients with low and high PCI.

## Patients and Methods

### Patients

From the prospectively-recorded database at our Comprehensive Cancer Centre (referral centre for all of South-West France), we identified all patients with peritoneal carcinomatosis of colorectal origins, synchronous or metachronous, with or without primary tumour resection and with or without liver or lung metastases, as long as they were eligible for surgical treatment, had complete cytoreductive peritoneal surgery (CCS0), and had received perioperative systemic chemotherapy. Patients had a minimum of one year follow-up and were included consecutively. Patients with CPM of other origins (especially appendiceal tumours) or incomplete cytoreduction after surgery were not considered for inclusion.

The Institut Bergonie internal review board (College de Recherche) approved this retrospective study using anonymized data. No informed consent was necessary. All patient files were discussed in a multidisciplinary team meeting with a medical oncologist, gastro-intestinal specialist, radiologist, surgeon and a specialist in nuclear medicine. All decisions were recorded prospectively in our database (Software Medlog 2000) and notified to the patient. All patients were clearly informed about the potential surgical risks of this aggressive procedure, the possibility of needing a derivative stoma or blood transfusion, and the uncertain duration of hospitalization in an intensive care unit. They all consented to receive treatment.

The pre-therapeutic assessment included a clinical examination, a computed tomography (CT) scan of the thorax, abdomen and pelvis, an 18FDG-PET-CT and a biological screening of main visceral functions. An anaesthesiologist evaluated all patients, selecting mostly ASA 1–2 patients for the surgical treatment. Patients with abundant ascites, usually associated with a high PCI or progressive under chemotherapy, were usually not operable and were thus not considered for CRS.

### Surgical procedure

Patients were positioned in lithotomy position to allow access to the rectum. A xypho-pubic laparotomy was carried out and a Thompson retractor installed (Landanger, France). Exploration of the peritoneum was conducted according to Sugarbaker’s peritoneal index (PCI). Patients were judged to be operable if the PCI score was less than 20-24/39.[12] A complete resection (CCS0) of all macroscopic disease within a maximum of 10 hours of surgery was recommended in order to limit morbidity and mortality. The operative field was washed hourly with a hot saline mix in order to limit the decrease of body temperature and to retrieve cellular debris. Liver metastases detected by intraoperative ultrasounds (BK Medical, Herlev, Denmark) were treated either by resection or intra-operative radiofrequency ablation [13]. Post-operative morbidity and mortality were registered according to the Dindo and Clavien classification.[14]

### Chemotherapy

As patients could be referred from other centres to our tertiary centre, there was variation in the type, mode and duration of chemotherapy administration. Some patients received preoperative chemotherapy, some post-operative and others both pre- and post-operative, providing that a total of at least 12 cycles of systemic chemotherapy were administered.

### Follow-up

Follow-up was made by clinical examination and a CT-scan (Thorax, abdomen and pelvis) with a dosage of carcino-embryon antigen (CEA) and carbohydrate antigen (CA 19.9) every 4 months for the first two years and then every 6 months for three subsequent years.

### Statistical analysis

Categorical variables are described in terms of frequency and percentages. The distributions of continuous variables are described with means or medians, standard error and range. The overall survival (OS) analyses took into account the delay from the CRS to the date of death (irrespective of cause) or to the last date the patient was known to be alive. Time until recurrence was defined as delay from the CRS to the date of first recurrence of CPM, or recurrence of CRC, or appearance of other metastases, whichever came first. Kaplan-Meier survival estimates are reported with their 95%CI and are compared with the log-rank test. Survivals were compared across subgroups of patients with and without other liver or lung metastases (LLM); and with a PCI index </≥7. Median follow-up was calculated using the reverse Kaplan-Meier method. The Stata v11.2 Texas USA statistical software was used for analyses.

## Results

### Patient characteristics

Between March 2003 and February 2012, 103 patients underwent surgical exploration in our institution for colorectal CPM. They were identified from our prospectively-maintained institutional database and the study population was defined as all patients undergoing a complete CCS0 cytoreductive surgery (50) with at least 12 cycles of systemic chemotherapy.

There were 24 men and 26 women with a mean age of 60.8 years (range: 22–82) (Table 1). Seven patients had a previous history of cancer other than colorectal (3 ovarian, 2 breast, 1 lymphoma, 1 prostate). For 44 patients the primary tumour was in the colon, six in the rectum. In addition to their peritoneal carcinomatosis, 26 patients also had other metastatic sites: 21 liver, 4 lung, 1 bone metastasis, 2 retroperitoneal lymph nodes metastases and 4 other sites (multiple sites possible: four patients had 1 other site and 1 patient had 2 other sites). In total, 23 patients had LLM. The remaining 3 patients with other non-visceral metastases are not included in the survival subgroup analyses.

**Table 1:** Patient characteristics.

	n
**Sex**	
Male	24
Female	26
**Other metastatic sites**	
No	24
Yes	26
Liver	21
Lung	4
Bone	1
Lymph nodes	2
Other visceral	4
**ASA score**	
**1**	8
**2**	38
**3**	4
**Delay of occurrence of carcinomatosis**	
Synchronous	22
Metachronous	28
**Peritoneal carcinomatosis index (Sugarbaker)**	
1–6	20
7–12	16
13–19	9
>19	4
Missing	1

Carcinomatosis was synchronous for 22 patients and metachronous for 28 with a median time of 29 months (range: 5–105) between primary tumour diagnosis and carcinomatosis. Seventeen patients received chemotherapy for their CPM before surgery. Thirty six patients received post-operative chemotherapy for the primary tumour. Forty five patients underwent surgery for the primary tumour before surgery for their CPM.

### Cytoreductive surgery

PCI ranged from 1 to 24 with a median of 8. Twenty patients had a PCI <7 and 29 ≥7 (one missing) (Table 2). Details of surgical procedures are reported in Table 3. Median length of surgery was 300 min (range: 90–790) with a median blood loss of 900mL (range: 0–4000). Nineteen patients underwent red blood cell transfusion. Liver metastases were found intra-operatively in 6 patients. Median length of stay was 15 days (range: 5–44). Median stay in the intensive care unit was 1 day (range: 0–4).

**Table 2:** PCI distribution.

Peritoneal carcinomatosis index (Sugarbaker)	Our series N (%)	Multicentre French study, (Elias et al, 2009[33]) N (%)
1–6	20 (40)	181 (37)
7–12	16 (32)	132 (28)
13–19	9 (18)	96 (21)
>19	4 (8)	69 (14)
missing	1 (2)	0

**Table 3:** Details of surgical procedures.

Procedures	n
Omentectomy	44
Colectomies (Right, left and transverse)	36
Small bowel resection	
Minor	15
<50%	6
Major	1
Rectum (part of Hudson procedure)	23
Hysterectomy	14
Oophorectomies	13
Bladder peritoneum (part of Hudson procedure)	29
Upper quadrant peritoectomies (diaphragm)	19
Glisson capsule cleaning	8
Stomach cleaning without gastrectomy	7
Parietal peritonectomies	20
Stomies	14
Liver procedures	
Right hepatectomy	1
Bisegmentectomy	4
Unisegmentectomy	7
Metastasectomy	13

### Complications

Complications are detailed in Table 4. There was no post-operative mortality. Twenty-seven complications were observed for 22 patients. Fifteen patients had a grade≥3 complication requiring percutaneous drainage and/or surgery and/or management in an intensive care unit. Eleven patients underwent reoperation for Grade 3b complications (1 wound abscess, 1 hemoperitoneum, 3 anastomotic leakage, 2 urinary fistulas, 2 biliary fistulas, 2 intra-abdominal abscesses).

**Table 4:** Complications for patients treated for colorectal peritoneal metastases with complete CRS and systemic chemotherapy only.

Grade (Dindo and Clavien)	n	%	Complication
1	2	4	Mood disorders
2	10	20	Urinary tract infection, dehydration, ileus, septicemia, pleural effusion
3a	3	6	Sub-phrenic abscess, colectasia
3b	11	22	Anastomotic leakage, intra-abdominal abscess, hemoperitoneum, urinary fistula
4a	1	2	Acute respiratory distress syndrome
4b	0	0	-
5	0	0	

### Post-operative chemotherapy

Thirty one patients received post-operative chemotherapy, in the majority a doublet cytotoxic regimen FOLFOX (17) or FOLFIRI (10) and Fluorouracil (1) (three missing), combined with a targeted therapy Cetuximab (15) or Bevacizumab (4) according to the k-Ras status (the “extended RAS” status was not yet available during this study). The median time to initiation of postoperative chemotherapy was 7 weeks (range: 2–13). Data about post-operative chemotherapy are missing for the other referred patients.

### Overall Survival

**Fig 1:**
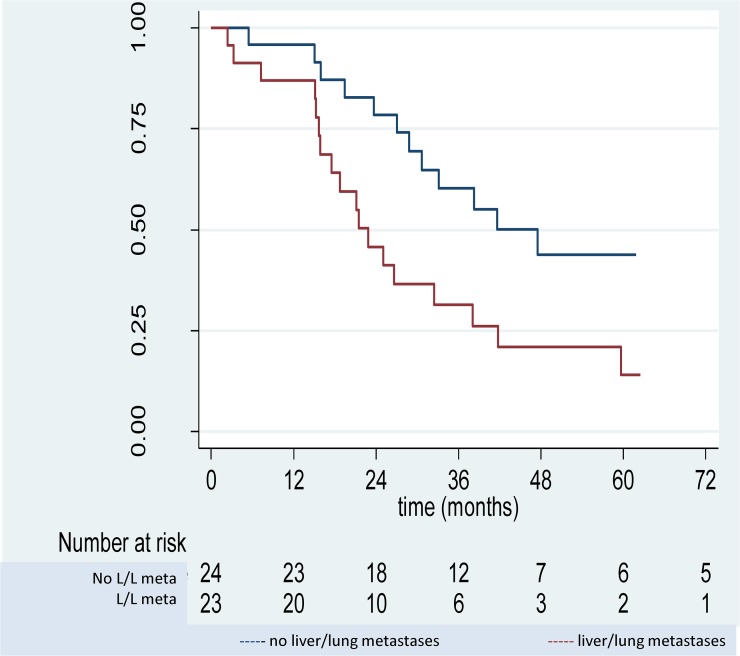
Overall survival for patients with colorectal peritoneal metastases treated by cytoreductive surgery and systemic chemotherapy according to presence of liver or lung metastases.

With a median follow-up of 62.5 months (95%CI 45.4–81.3), 15 patients were alive at last news, 33 patients were dead, and 2 were lost to follow-up. Median survival was 32.4 months (21.5–41.7). Three-year OS was 45.5% (0.31–0.59) and 5-year OS 29.64% (0.17–0.44) (Fig 1). Presence of LLM was significantly associated with poorer prognosis, with survival at 5 years of 13.95% (95%CI 2.9–33.6) versus. 43.87% (22.2–63.7) when no metastases present (*P* = 0.018) (Fig 1). For patients with no other metastases, median OS was 47.5 (95%CI 28.8- not reached) months, much longer than for patients with LLM at 22.8 (95%CI 15.7–38.1) months) (excluding three patients with other non-visceral metastases). Prognosis was also correlated with the PCI. Median survival was longer for lower PCI (1–6) vs. higher PCI (7–24), (47.5 months [95%CI 30.7-nc] vs. 22.9 months [16.7–33.1], *P* = 0.047) and 3- and 5-year OS were better for lower PCI than for higher (respectively 67.39% (95%CI 41.1–83.9) versus 32.33% (16.3–49.6) and 35.94% (12.6–60.3) versus 25.14% (11.1–42.0)).

### Progression-free survival

**Fig 2:**
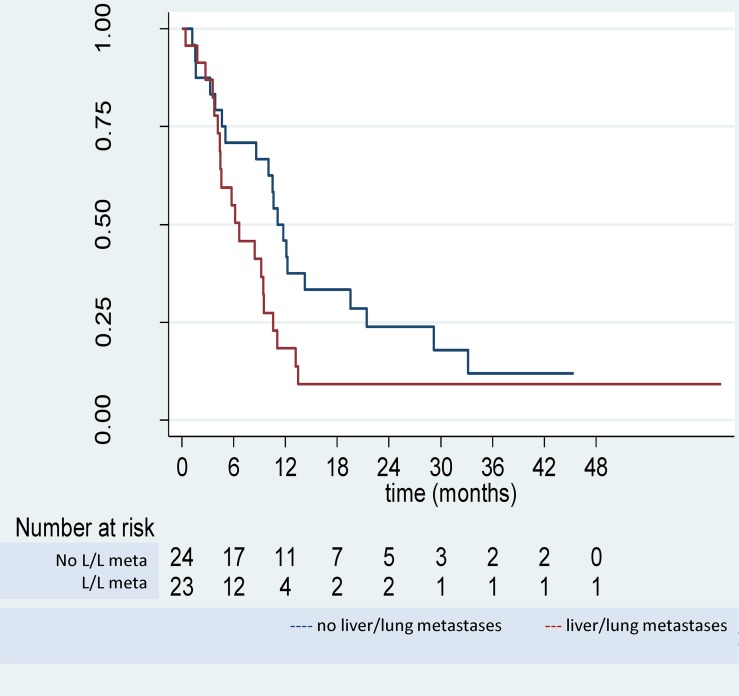
Progression-free survival for patients with colorectal peritoneal metastases treated by cytoreductive surgery and systemic chemotherapy according to presence of liver or lung metastases.

Forty two patients had a recurrence. This included 34 patients with recurrence of their CPM (12 CPM only), and 8 patients with extraperitoneal recurrence only. Median progression-free survival was 9.5 months (95%CI 6.2–11.1) (Fig 2). For patients with no LLM, a favourable non-significant trend was observed for PFS with a median of 11.1 months (95%CI 5.0–19.5) vs. 6.6 months (4.1–9.5) when LLM present (*P* = 0.067). Median PFS was 11.8 months (4.4–33.2) for patients with PCI of 1–6 and 6.6 months (4.6–9.5) for PCI ≥7.

## Discussion

Common sense and personal experience cannot replace randomized trial data in modern medicine. When treatment strategies are decided upon without empirical data, severe disappointments may appear some years later in clinical situations, especially if the innovation was considered so evident that randomized data were not considered necessary to adopt the practice [10]. On the other hand, one randomized trial is not enough evidence to standardise a practice approach. The entire clinical rational of HIPEC was based on the trial by Zoetmulder et al[7] published in 2003. This trial asserted the superiority of CRS + HIPEC + chemotherapy versus chemotherapy alone (5FU + folinic acid as a bolus) for patients with CPM with no other metastases. A median survival of 22.3 months was observed for the treatment group versus 12.6 for the control group. It should be noted that 18/105 patients had appendiceal tumours which have a different histological type with a better prognosis, and which are now generally excluded from reports on CPM. In the subset analysis, appendiceal tumours appeared to benefit the most from the surgical treatment compared to the pure colorectal group. By the authors’ own admittance, this trial cannot disentangle the possible effects of HIPEC from the effectiveness of CRS. Moreover, the results of this trial are no longer relevant given the new standards of palliative chemotherapy for metastatic CRC which offer much improved OS of at least 24 months,[15] approaching 30 months recently.[16] The late results of this trial published in 2008[17] are of particular interest, although they are rarely discussed in the literature. There were 5 patients alive in the HIPEC arm, 2 with and 3 without disease and 4 patients alive in the standard arm, 2 with and 2 without disease. The median disease-specific survival was 12.6 months in the control group versus 22.2 months in the HIPEC arm but clearly, in this group, only the patients having a R1 surgery showed a real survival advantage (5-year OS at 45%). Once again, the most legitimate conclusion that can be drawn from this article is that, from the CRS/Hyperthemia/Peritoneal chemotherapy triplet, complete CRS is the only one for which a certain degree of scientific evidence has been reached. This hypothesis was later confirmed by a retrospective review of 523 patients over 17 years where having an HIPEC was not identified as a prognostic factor.[18]

Reasoning by analogy with CR liver metastases, in our practice in 2000 we decided to consider the whole peritoneum as an organ for which metastatic dissemination can be mainly addressed by systemic chemotherapy and complete surgery. In the literature, we found only 3 studies reporting on patients with peritoneal metastases without HIPEC or EPIC. A study by Elias et al[19] was stopped for poor accrual but appeared to show no differences in the 2-year OS rate (60% in the two groups). A retrospective study by Scaringi et al[20] with 27 patients treated by CRS without HIPEC had a median OS of 15.8 months for the CCR0-CCR1 patients and a very high mortality rate (7.4%). In a prospective series of 50 patients treated by surgery plus systemic chemotherapy the median OS was only 17.3 months, but only 7 patients had an R0/R1 surgery. Our series is the first to be clearly dedicated to a complete cytoreductive surgery plus systemic chemotherapy.

Our median operation duration was 5 hours compared to between 5[7,21] and 8 hours[7] previously reported with HIPEC. Our median blood loss was 0.9 L versus 1 to 5L.[21–23] These factors both indicate a lower risk of dissemination and consequently lower risk of local recurrence. The duration of hospital and intensive care unit stays appear shorter in this series than in others: 15 days hospitalisation versus between 15[24] and 29 days,[7] and 1 day intensive care versus 2 to 7 days.[21–23] We also observed no mortality, compared to 0–12% reported in a literature review after HIPEC[25] even if, more recently, the mortality rate in the institutions regarded as tertiary high-volume centres, has been reported as between 0.9 and 5.8%.[22]

Our morbidity observed for 22 of 50 patients is not dissimilar to rates reported in other series (9.6%[26] to 77%[27]), even though 44% of complications taken into account in our series were Grade 1 and 2 complications generally not included in older series. Eleven patients had to be re-operated in the short-term because of complications. This high number may be explained by the fact that complications were operated on quickly to avoid grade 4 and 5 morbidity (which was experienced by only one patient in our series).

We observed a median OS of 32.4 months. This is within the range in the literature for comparable R0 patients receiving CRS and HIPEC treatment of 14[28] to 62[29] months. The present series had 1-year and 3-year OS of 89 and 45.8% respectively. This is also well within the ranges in the literature for R0 patients receiving HIPEC for 1-year survival: 55%[30] to 100%[31] and for 3-year survival: 37%[29] to 62%.[32] The variation in results from the literature may be explained by the heterogeneity of the series reported, for example the range of PCI included and the frequency of liver metastases. If we compare our series to the French retrospective analysis,[33] we can observe that both series have approximately the same ranges for PCI distribution (Table 2). For the rate of operated liver metastases, we report a higher rate of 42 per cent versus 17 per cent.[33] Three-year OS was 45.8% compared to 41%.[33] In a recent publication, 102 patients achieved a complete CRS, median PCI was 11, 22.5% had their liver metastases resected and their OS noted on the survival curve was roughly 46% at 3 years,[34] and 35% at five years. When comparing our results to the literature, it should also be noted that a lot of series have reported rates lower than 17% of associated LM.[7,18,24,30,35]

For patients without LLM, median OS was 47.5 months, longer than for patients with LLM (22.8 months). Note that the comparison was possible as median PCI’s were relatively similar across the two groups. This result is slightly lower than that of Elias et al reporting OS of 62.7 months for a selective group of patients with CRS and no liver metastases, treated by CRS + HIPEC with chemotherapy.[12] However, it is not supported by other contributions reporting no differences in survival when liver metastases were small, less than 3 and resectable.[36,37] Kianmanesh et al.[37] observed a median OS of 36 months for non-metastatic patients versus 35.3 months for patients with resection of liver metastases, however only 70% of patients were R0. Chua et al.[36] reported a median OS of 36 months with no survival differences for patients with or without liver metastases.

Recurrence of CPM is a frequent and problematic issue. When a new complete resection is possible, a second procedure with CRS + HIPEC has been recommended to improve survival.[38,39] It may be interesting to discuss if a routine second look should be added to the strategy in order to increase OS.

The heterogeneity in the timing, duration and type of chemotherapy used represents a limitation in our retrospective series as this is a significant confounding factor potentially affecting outcomes. The high level of missing information comes specifically from the fact that many patients were referred to our institute after receiving first-line chemotherapy and the information on chemotherapy administration is not available. However, all patients received at least 12 cycles, before and/or after CRS, like the majority of patients included in HIPEC series. Without surgery, modern chemotherapies plus targeted therapies offer median OS of 24 months[40] and recently 29-month survival was reported in the CALGB/SWOG 80405 study. Even if progress of palliative chemotherapy may reduce the need for surgical treatment in the future, it should be stressed that as surgery is always accompanied by chemotherapy, each treatment will benefit from the progress of the other one.

Awaiting the results of the Prodige randomized trial (NCT00769405), this study, albeit retrospective as the rest of the available literature on HIPEC, brings evidence that complete CRS + chemotherapy only is a robust hypothesis to become the validated treatment standard for resectable colorectal peritoneal metastases.
